# Rab coupling protein mediated endosomal recycling of N-cadherin influences cell motility

**DOI:** 10.18632/oncotarget.10513

**Published:** 2016-07-09

**Authors:** Andrew J. Lindsay, Mary W. McCaffrey

**Affiliations:** ^1^ Molecular Cell Biology Laboratory, School of Biochemistry and Cell Biology, Biosciences Institute, University College Cork, Cork, Ireland

**Keywords:** RCP, migration, epithelial-mesenchymal transition, N-cadherin, endosomal recycling

## Abstract

Rab coupling protein (RCP) is a Rab GTPase effector that functions in endosomal recycling. The RCP gene is frequently amplified in breast cancer, leading to increased cancer aggressiveness. Furthermore, RCP enhances the motility of ovarian cancer cells by coordinating the recycling of α5β1 integrin and EGF receptor to the leading edge of migrating cells. Here we report that RCP also influences the motility of lung adenocarcinoma cells. Knockdown of RCP inhibits the motility of A549 cells in 2D and 3D migration assays, while its overexpression enhances migration in these assays. Depletion of RCP leads to a reduction in N-cadherin protein levels, which could be restored with lysosomal inhibitors. Trafficking assays revealed that RCP knockdown inhibits the return of endocytosed N-cadherin to the cell surface. We propose that RCP regulates the endosomal recycling of N-cadherin, and in its absence N-cadherin is diverted to the degradative pathway. The increased aggressiveness of tumour cells that overexpress RCP may be due to biased recycling of N-cadherin in metastatic cancer cells.

## INTRODUCTION

The endosomal recycling pathway mediates the transport of endocytosed proteins back to the plasma membrane, and it is emerging that aberrant expression of key regulators of this pathway can lead to increased aggressiveness in a wide range of cancers [1, 2]. Rab11 regulates cargo transport from the endosomal recycling compartment (ERC) to the plasma membrane. Rab25, an epithelial-specific isoform of the Rab11 subfamily, has been reported to act as both an oncogene and tumour suppressor gene in various cancers [3]. RCP is a Rab11 effector protein encoded by a gene that maps to chromosome 8p11-12, a region that is frequently amplified in cancer [4]. RCP has been found to be a breast cancer promoting gene and its overexpression significantly correlates with aggressive breast cancer [5]. Furthermore, RCP mediates the recycling of α_5_β_1_ integrin and the EGF receptor to the leading edge of migrating A2780 ovarian cancer cells [6], and this recycling is enhanced by mutant p53 [7].

RCP belongs to a family of proteins whose members are characterised by a highly conserved ∼20 amino acid Rab-binding domain (RBD) at their C-termini that mediates their interaction with Rabs. There are five key members of this Rab11-Family of Interacting Proteins (FIPs), and the FIPs can be subdivided into two classes. The class I FIPs possess a C2 domain at their amino terminus which preferentially binds to phosphatidic acid and PI(3, 4, 5) P_3_ at the plasma membrane [8, 9]. The class II FIPs lack a C2 domain but possess calcium-binding EF-hand motifs. In addition to Rab11, the class I FIPs can also bind to Rab14 [10, 11], which localises to early endosomes, the endosomal recycling compartment and the Golgi [12]. Rab14 has been reported to play a role in N-cadherin junctional complex formation by stimulating the transport of the ADAM10 metalloproteinase to the plasma membrane of A549 lung adenocarcinoma cells [13]. ADAM10 cleaves N-cadherin at the plasma membrane resulting in the shedding of its ectodomain. The Barr group found that depletion of Rab14 trapped ADAM10 in an intermediate recycling compartment, preventing it reaching the plasma membrane and leading to a dramatic increase of full-length N-cadherin at the cell surface. Rab14 and RCP have recently been implicated in the trafficking of the HIV-1 Env glycoprotein to the plasma membrane in infected cells [14].

Given the previously described role for RCP in promoting the migration of breast and ovarian cancer cells, and the fact that the 8p11-12 amplicon has been frequently observed in other cancers [15–18], we decided to investigate whether RCP has a similar role in non-small cell lung cancer (NSCLC) cells. Lung cancer is highly aggressive and is the leading cause of cancer deaths worldwide, with an overall 5-year survival rate of less than 13%. We found that RCP knockdown resulted in a reduction in the motility of A549 cells, whereas overexpression of wild-type RCP led to increased motility. RCP knockdown resulted in a loss of polarisation of the Golgi complex and a reduction in the levels of N-cadherin protein. Trafficking assays demonstrated that RCP plays an important role in the recycling of endocytosed N-cadherin to the cell surface. Treatment of RCP-depleted cells with lysosomal inhibitors restored N-cadherin levels suggesting that in the absence of RCP, internalised N-cadherin is diverted to the degradative pathway where it is degraded in lysosomes.

## RESULTS

### RCP is required for the migration of non-small cell lung cancer cells

**Figure 1:**
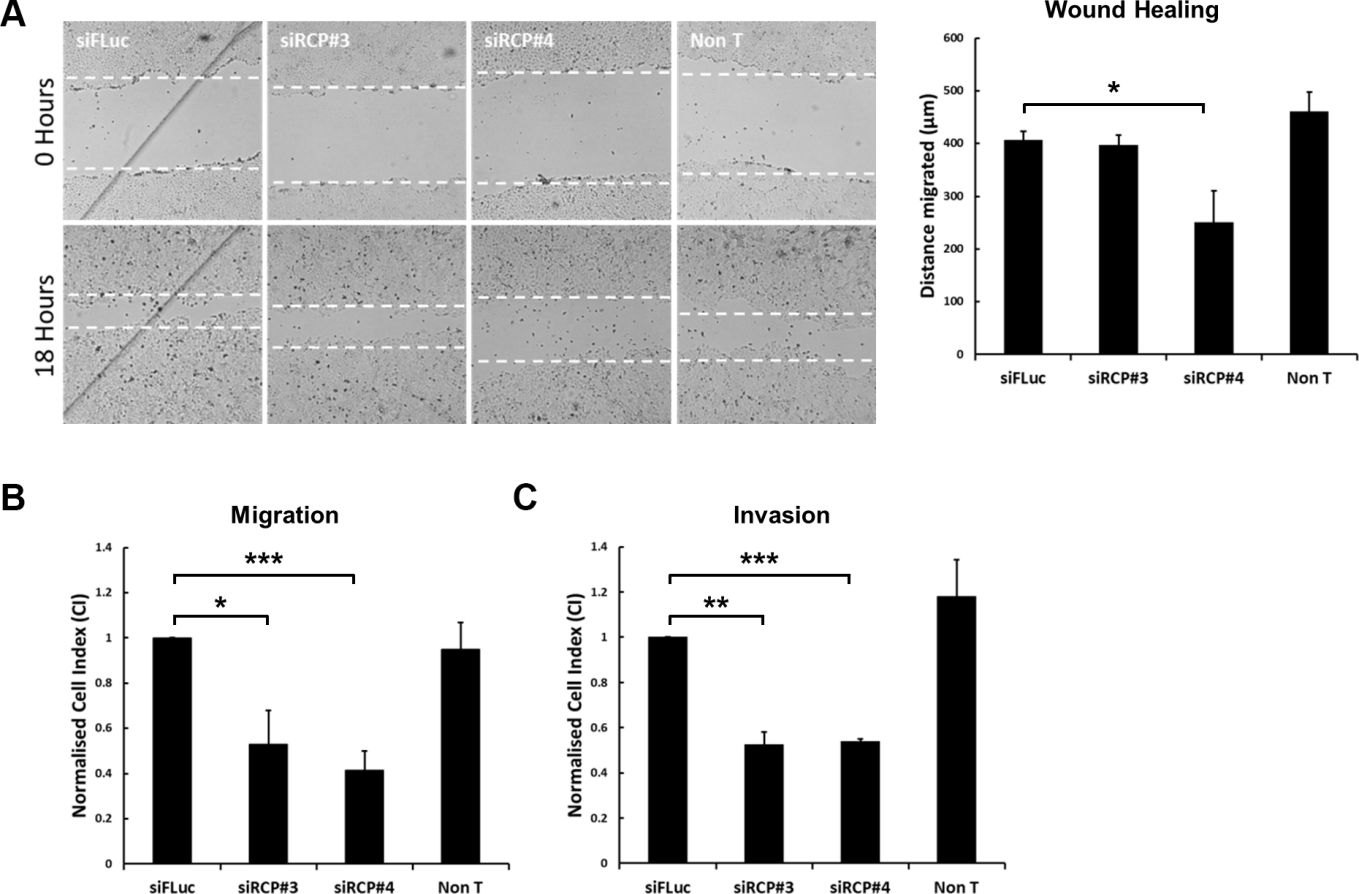
Downregulation of RCP inhibits cell motility (**A**) A549 cells in 24-well plates were transfected with a control siRNA or two siRNA duplexes targeting RCP. 72 hours post-transfection the cell monolayers were wounded and brightfield images recorded. The cells were returned to 37^°^C for 18 hours and imaged again. The distance migrated by the wound front is plotted in the bar graph. Error bars indicate the standard error of the means (^*^p < 0.05; *n* = 3). (**B**) A549 cells were transfected with the indicated siRNA duplexes for 72 hours, detached and seeded in duplicate on CIM-16 Transwell plates and subjected to real-time migration assays (xCelligence). The histogram depicts the Normalised Cell Index (CI) after 24 hours of migration. Error bars indicate the standard error of the means (^*^p < 0.05, ^***^p < 0.001; *n* = 4). (**C**) A549 cells transfected with the indicated siRNA duplexes for 72 hours, were detached and seeded, in duplicate, on CIM-16 Transwell plates that had been coated with Matrigel. The histogram depicts the Normalised Cell Index (CI) after 48 hours of migration. Error bars indicate the standard error of the means (^**^p < 0.01, ^***^p < 0.001; *n* = 3).

To investigate if RCP serves a role in the migration of NSCLC cells, A549 cells were transfected with a control siRNA, that is complementary to the Firefly luciferase mRNA (siFLuc), or two separate siRNA duplexes that target RCP (siRCP#3 and siRCP#4). 72 hours post-transfection, when the cells have reached confluency, they were scratched with a plastic pipette tip and returned to 37^°^C. After 18 hours the distance that the cells had migrated into the wound was measured. We typically achieved ∼ 90% knockdown of RCP under these conditions (Supplementary Figure S1A, S1B). A549 cells transfected with siRCP#4 displayed a 40% reduction of migration into the wound (Figure 1A). However, siRCP#3 had no significant effect (Figure 1A).

Given the inconclusive results obtained from the scratch-wound assays, and previous observations that Rab25 influences cell motility in 3D migration assays but not in 2D assays [19], we proceeded to investigate whether RCP depletion affected the motility of A549 cells in 3D migration assays. We used a real-time impedance-based assay (xCelligence) to monitor the migration of cells through a semipermeable membrane containing 8 μm pores. In this migration assay, both RCP targeting siRNA duplexes significantly inhibited the migration of the transfected cells (Figure 1B). Inhibition was also observed in invasion assays in which the transfected cells were seeded on top of a layer of Matrigel and the cells had to penetrate through this reconstituted basement membrane before they reach the semipermeable barrier (Figure 1C).

**Figure 2:**
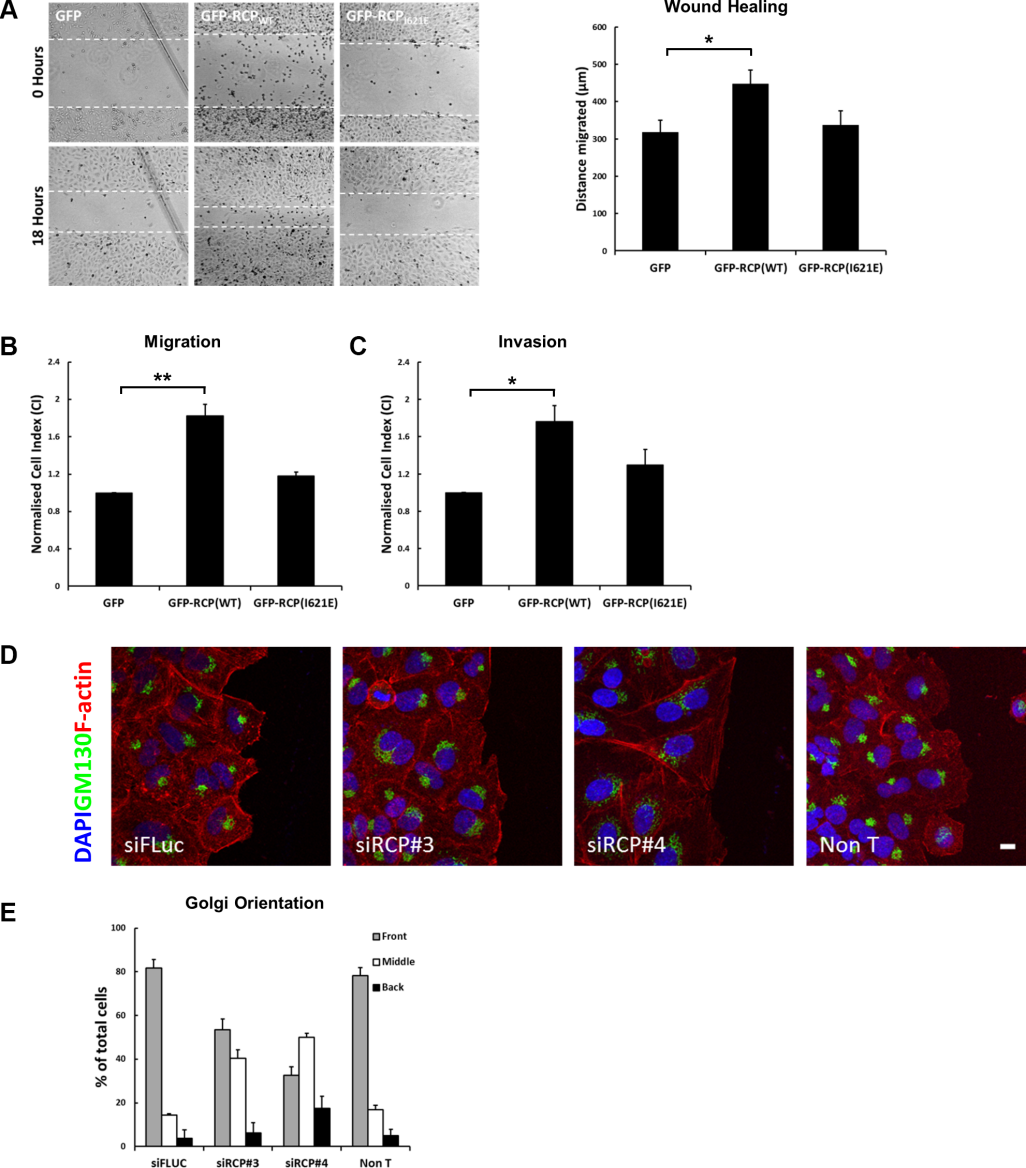
Overexpression of RCP promotes cell motility (**A**) Monolayers of A549 cells induced to express GFP, GFP-RCP_WT_, or GFP-RCP_I621E_ for 24 hours were wounded and bright-field images recorded. The cells were returned to 37^°^C for 18 hours and imaged again. The distance migrated by the wound front is plotted in the bar graph. Error bars indicate the standard error of the means (^*^p < 0.05, *n* = 3). (**B**) A549 cells expressing the indicated GFP-fusion constructs were seeded, in duplicate, on CIM-16 Transwell plates. The histogram depicts the Normalised Cell Index (CI) after 24 hours of migration. Error bars indicate the standard error of the means (^**^p < 0.01; *n* = 3). (**C**) A549 cells expressing the indicated GFP-fusion constructs were seeded, in duplicate, on CIM-16 Transwell plates that had been coated with Matrigel. The histogram depicts the Normalised Cell Index (CI) after 48 hours of migration. Error bars indicate the standard error of the means (^*^p < 0.05; *n* = 5). (**D**–**E**) A549 cells transfected with the indicated siRNA duplexes for 72 hours, were wounded and allowed to recover at 37^°^C for 5 hours. The cells were fixed and labelled with DAPI (blue), anti-Texas Red-phalloidin (red), and the Golgi complex was labelled with anti-GM130 (green). Bar, 10 μm (D). The localisation of the Golgi in > 100 cells, from 3 independent experiments, is depicted in the bar graph (E).

We next set out to investigate the effect of RCP overexpression in these cell motility assays. To this end, A549 cell lines stably transfected with plasmids expressing green-fluorescent protein (GFP) alone, GFP fused wild-type RCP (GFP-RCP_WT_), or a mutant of RCP with a single amino acid change in its RBD that abolishes the interaction with Rab11 and Rab14 (GFP-RCP_I621E_) [11], were generated. Expression of the fusion protein is induced by supplementing the growth medium with sodium butyrate 24 hours prior to the experiment (Supplementary Figure S1C, S1D). Quantification revealed that 5mM sodium butyrate induced levels of GFP-RCP_WT_ and GFP-RCP_I621E_ expression of 3.1 ± 0.02 and 2.5 ± 0.14 fold over that of endogenous RCP, respectively (Supplementary Figure S1E). Overexpression of wild-type RCP increased the motility of A549 cells in the scratch-wound (Figure 2A), migration (Figure 2B), and invasion assays (Figure 2C), whereas the cell line expressing RCP_I621E_ migrated at the same rate as the control cells expressing GFP alone (Figure 2A–2C). To determine if the reduction in cell motility observed upon siRNA-mediated depletion of endogenous RCP could be rescued by overexpression of GFP-RCP, we transfected the stable cell lines with an siRNA that targeted the 5′ untranslated region of RCP (siRCP#5). Induction of GFP-RCP_WT_, but not GFP-RCP_I621E_, in cells transfected with siRCP#5 rescued the inhibitory effect, in both wound healing and cell migration assays (Supplementary Figure S2A, S2B). This rules out the possibility that the suppression of cell motility observed when RCP is downregulated is due to off-target effects of the siRNA complexes.

Taken together, these results demonstrate that RCP plays an important role in regulating the migration of A549 lung adenocarcinoma cells, and agrees with similar findings in breast and ovarian cancer cells [5, 6].

To investigate if defective polarisation of RCP-depleted cells may account for the inhibition in migration, monolayers of A549 cells transfected with control and RCP siRNA duplexes were wounded and allowed to recover for 5 hours. The localisation of the Golgi complex in cells at the wound edge was determined by immunofluorescent labelling of the GM130 *cis*-Golgi marker. Cells orient their Golgi to face the direction of migration in wounded cells [20], and in ∼ 80% of control cells the Golgi complex was oriented in front of the nucleus facing the wound edge (Figure 2D, 2E). In contrast, depletion of RCP resulted in a considerable reduction in the proportion of cells with wound-oriented Golgi complexes (siRCP#3 = 53.5 ± 5.0%; siRCP#4 = 32.6 ± 3.8%), indicating that an inability to establish an axis of polarity with respect to the wound edge may contribute to the migration defects.

### RCP regulates cadherin protein levels

**Figure 3:**
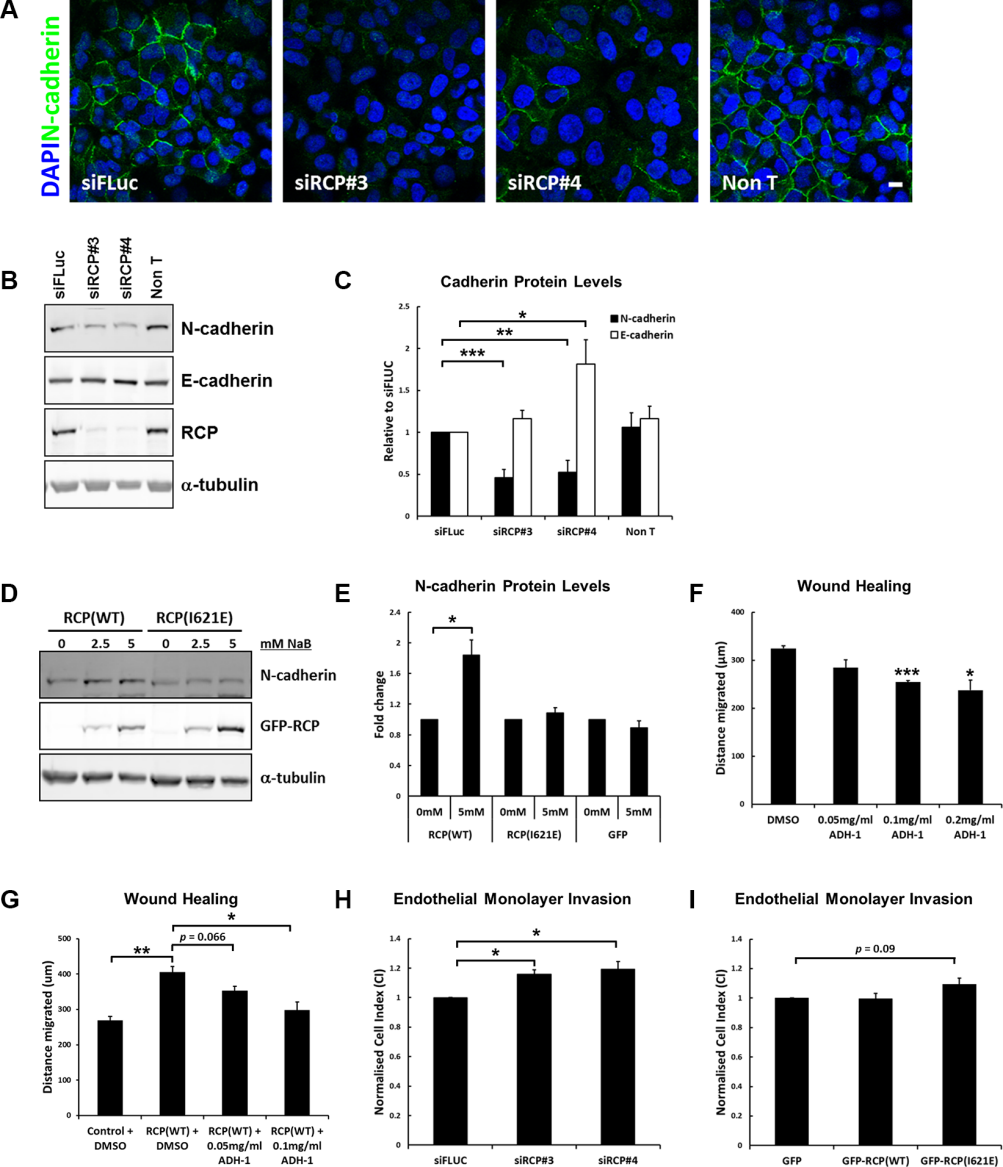
RCP regulates cadherin protein levels (**A**) A549 cells transfected with the indicated siRNA duplexes for 72 hours, fixed and labelled with DAPI and anti-N-cadherin. Bar, 10 μm. (**B**) A549 cells transfected with the indicated siRNA duplexes were lysed after 72 hours and Western blotted with the indicated antibodies. α-tubulin was used as a loading control. (**C**) Histogram represents the level of N-cadherin (black) and E-cadherin (white) protein in lysates from cells transfected for 72 hours with the indicated siRNA duplexes. Values are normalised to siFLuc (^*^p < 0.05, ^**^p < 0.01, ^***^p < 0.001; *n* = 6). (**D**) Representative Western blots of lysates from A549 GFP-RCP_WT_ and GFP-RCP_I621E_ stable cell lines treated with the indicated concentrations of sodium butyrate for 24 hours. (**E**) Level of N-cadherin protein in lysates from the indicated A549 stable cell lines treated +/− 5 mM sodium butyrate for 24 hours (^*^p < 0.05; *n* = 3). (**F**) A549 cells grown to confluency were wounded and allowed to recover for 18 hours at 37^°^C in the presence of DMSO (vehicle control) or the indicated concentration of ADH-1 (Exherin). The distance migrated by the wound front is plotted in the bar graph. Error bars indicate the standard error of the means (^*^p < 0.05, ^***^p < 0.001; *n* = 3). (**G**) A549 or A549 GFP-RCP_WT_ cells were grown to confluency, wounded and allowed to recover for 18 hours at 37^°^C in the presence of DMSO (vehicle control) or the indicated concentration of ADH-1 (Exherin). The cells were treated for a total of 24 hours with 5mM sodium butyrate to induce expression of the fusion protein (6 hours pre-wounding and 18 hours post-wounding). The distance migrated by the wound front is plotted in the bar graph. Error bars indicate the standard error of the means (^*^p < 0.05, ^**^p < 0.01; *n* = 3). (**H**) A549 cells transfected with the indicated siRNA duplexes were detached and seeded, in duplicate, on top of a monolayer of HUVECs grown in an E-16 plate. The histogram indicates the Normalised Cell Index, normalised to siFLUC, 7 hours after addition of the A549 cells. Error bars indicate the standard error of the means (^*^p < 0.05; *n* = 3). (**I**) A549 cells expressing the indicated GFP-fusion protein were seeded, in duplicate, on top of a monolayer of HUVECs grown in an E-16 plate. The histogram indicates the Normalised Cell Index, normalised to GFP, 7 hours after addition of the A549 cells. Error bars indicate the standard error of the means (*n* = 3).

Rab14 was recently found to play a role in controlling N-cadherin protein levels, by regulating the transport of the ADAM10 metalloproteinase to the plasma membrane where it cleaves N-cadherin [13]. Given that Rab14 interacts with RCP, and N-cadherin has been found to promote the motility and invasion of tumour cell lines [21–23], we investigated whether RCP knockdown could also affect N-cadherin protein levels. We observed a striking reduction in the amount of N-cadherin at the plasma membrane by immunofluorescence (Figure 3A), and quantification by Western blot revealed an approximately 50% reduction in total N-cadherin protein levels in RCP-depleted cells (Figure 3B, 3C). Rab11 has a well characterised role in the trafficking of E-cadherin [24, 25]; therefore we investigated whether E-cadherin levels were also reduced under the same conditions. Surprisingly, we found that transfection with RCP siRNA resulted in an increase of E-cadherin levels relative to siFLuc transfected cells, or a non-transfected control (Figure 3B, 3C).

A greater than 80% increase in N-cadherin protein levels was observed in the GFP-RCP_WT_ stable cell line treated with 5mM sodium butyrate for 24 hours (to induce expression of the fusion protein), but there was no such increase in the GFP, and GFP-RCP_I621E_ cell lines (Figure 3D, 3E). E-cadherin levels were not significantly altered in all of these cell lines. The RCP gene is frequently amplified in cancer (Supplementary Figure S3A), and analysis of data from the TCGA collective Lung Squamous Cell Carcinoma panel (TCGA, provisional) revealed that the expression of N-cadherin protein is elevated in tumours in which the RCP gene is amplified (Supplementary Figure S3B), which is in agreement with our *in vitro* data.

E-cadherin and N-cadherin are important factors in the invasion and growth of epithelial cancer cells, with E-cadherin acting as a ‘brake’ on invasion while N-cadherin promotes invasion and metastasis [26, 27]. Thus, the effect of altering the expression of RCP on the motility of A549 cells is likely to be due to the concomitant change in the levels of E- and N- cadherin. To investigate whether the enhanced motility of A549 cells expressing GFP-RCP_WT_ may be due to the increased level of N-cadherin in these cells, we performed wound healing assays in the presence or absence of the N-cadherin inhibitor Exherin (ADH-1). Exherin is a cyclic pentapeptide that acts as a specific N-cadherin antagonist [28]. Addition of Exherin to the culture medium in normal wounded A549 cells leads to a dose-dependent delay in the closure of the wound (Figure 3F), and Exherin suppressed the enhanced migration of the GFP-RCP_WT_ -overexpressing A549 cells (Figure 3G). This result confirms that the higher level of N-cadherin in these cells is an important factor contributing to their increased motility.

The dissemination of metastatic cancer cells requires the degradation of basement membrane, adhesion to endothelial cells of the vasculature, retraction of endothelial junctions, and finally invasion of the tumour cell into the blood stream in order to spread to distant sites in the host. Once in the circulation, cancer cells can then adhere to capillary walls and extravasate into the surrounding tissue to form metastatic tumours [29]. N-cadherin is expressed on the surface of endothelial cells and during intravasation it is thought to act as a receptor for the attachment of cancer cells that are overexpressing N-cadherin. To further measure the role that RCP plays in cell invasiveness, the effect of A549 cells on the permeability of a barrier of endothelial cells was monitored. Human umbilical vein endothelial cells (HUVECs) were seeded onto gold electrodes that line the bottom surface of a microplate attached to the xCelligence instrument. This allows the real-time monitoring of changes of electrical resistance as a cell monolayer, with intact cell-cell junctions, forms. The impedance values are translated into cell indexes (CI) that increase over time until they reach a plateau, indicating that a tight junctional barrier has been obtained. The CI values for HUVEC cells typically reach a plateau after 19–21 hours. At this point A549 cells, that had been transfected for 72 hours with control or RCP siRNA, are added to the HUVEC monolayer and CI is monitored for a further 7 hours. Disruption of the barrier integrity due to the retraction of the endothelial junctions by the tumour cells leads to a decrease in the CI. A549 cells depleted of RCP were less efficient at disrupting the integrity of endothelial junctions, as indicated by a higher cell index for the HUVEC monolayer seeded for 7 hours with A549 cells transfected with siRNAs targeting RCP (siRCP#3 CI = +16 ± 2.9%; siRCP#4 CI = +19.2 ± 5.2%) (Figure 3H). The A549 cells stably overexpressing GFP-RCP_WT_ did not promote increased endothelial junction disruption when compared to cells expressing GFP alone, however the GFP-RCP_I621E_ cells displayed ∼ 10% inhibition in disruption of junctional integrity (Figure 3I).

### Depletion of RCP disrupts the cadherin switch during epithelial-mesenchymal transition

**Figure 4:**
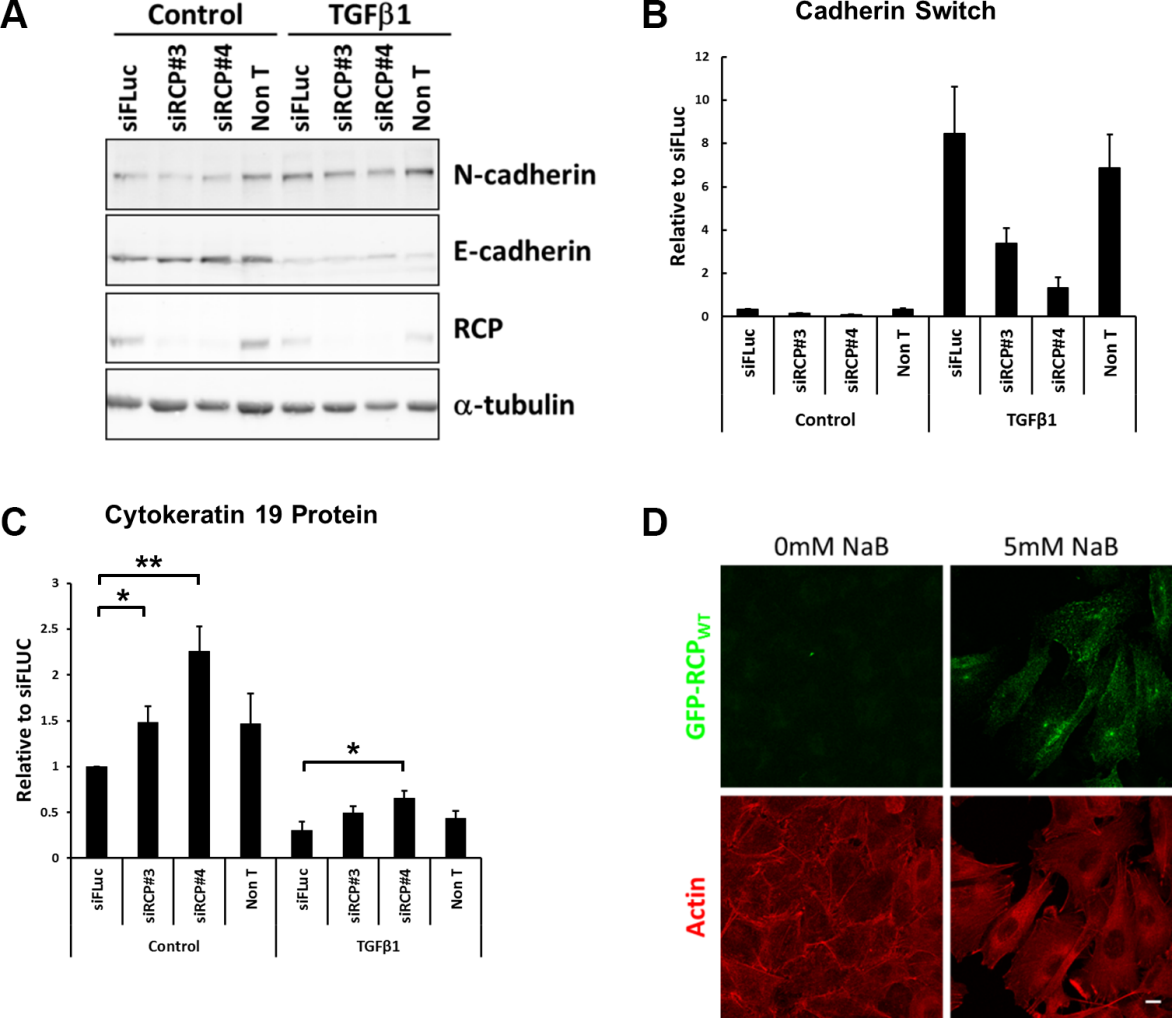
RCP knockdown disrupts the cadherin switch during EMT (**A**) Representative Western blots of lysates from A549 cells were transfected with the indicated siRNA duplexes for 24 hours and then treated +/− 5 ng/ml TGFβ1 for a further 48 hours. α-tubulin was used as a loading control. (**B**) Histogram indicating the ratio of E-cadherin to N-cadherin protein in A549 cells transfected with the indicated siRNA duplexes for 24 hours and treated +/− 5ng/ml TGFβ1 for a further 48 hours. Values are normalised to siFLuc Control (*n* = 3). (**C**) Histogram indicating the levels of the cytokeratin 19 intermediate filament protein in A549 cells transfected with the indicated siRNA duplexes for 24 hours and treated +/− 5 ng/ml TGFβ1 for a further 48 hours. Values are normalised to siFLuc Control (^*^p < 0.05, ^**^p < 0.01; *n* = 5). (**D**) Fluorescence images of the A549 GFP-RCP_WT_ stable cell line treated +/− 5 mM sodium butyrate for 24 hours prior to fixation and labelling with Texas Red-phalloidin (red). Bar, 10 μm.

The ratio of E-cadherin to N-cadherin is important to the process of epithelial-mesenchymal transition (EMT), the trans-differentiation of epithelial cells into motile mesenchymal cells. A hallmark of EMT is the downregulation of E-cadherin, leading to the destabilisation of adherens junctions, and a corresponding increase in expression of N-cadherin. This ‘cadherin switch’ facilitates cell migration and invasion [30]. A549 cells can be induced to undergo EMT by treatment with recombinant human TGFβ1 ligand for 48 hours, however RCP knockdown dampens the cadherin switch (i.e. N-cadherin protein level/E-cadherin protein level) by 2.5- to 6- fold under these conditions (Figure 4A, 4B). EMT is accompanied by the up- or down- regulation of various transcription factors, cytoskeletal proteins, signalling proteins and miRNA species. We looked at a number of these and found that there were elevated levels of β-catenin when RCP was knocked down (Supplementary Figure S4E), but no effect on the TWIST and TCF4 transcription factors (Supplementary Figure S4D, S4F). The intermediate filament proteins cytokeratin 19 and vimentin are down- and up- regulated, respectively, during EMT. We detected no difference in vimentin levels when RCP was depleted (not shown), however the level of cytokeratin 19 was higher in the RCP-knockdown cells when compared to controls (Figure 4C). These results suggest that RCP knockdown maintains A549 cells in an ‘epithelial-like’ state by affecting the levels of a subset of EMT markers.

EMT is also accompanied by a morphological change, and comparison of sodium butyrate treated and untreated GFP-RCP_WT_ stable cells by fluorescence microscopy revealed that the untreated cells, in which RCP_WT_ is not overexpressed, maintained their ‘cobblestone’ appearance characteristic of epithelial cells. In contrast, induction of GFP-RCP_WT_ led to the cells adopting a more mesenchymal morphology i.e. the cells had lost defined cell-cell contacts, became more elongated, and developed prominent stress fibres (Figure 4D), indicating that upregulation of RCP expression is sufficient to induce the cells to adopt a mesenchymal morphology.

### RCP regulates the recycling of N-cadherin

The reduction in N-cadherin levels upon RCP downregulation may be due to one of a number of factors; (i) RCP knockdown affects N-cadherin mRNA transcription, (ii) RCP knockdown leads to increased N-cadherin shedding at the plasma membrane, (iii) RCP knockdown promotes proteasomal degradation of N-cadherin, or (iv) RCP knockdown promotes the lysosomal degradation of N-cadherin.

Quantitative RT-PCR revealed that RCP depletion did not consistently affect the levels of N-cadherin mRNA (Supplementary Figure S4A, S4B), ruling out the possibility that RCP regulates N-cadherin expression at the transcriptional level. Moreover, while lung squamous cell tumours in which the RCP gene has been amplified show increased expression of N-cadherin protein (Supplementary Figure S3B), the N-cadherin mRNA levels are unaffected in these tumours (Supplementary Figure S3C). This provides further evidence that RCP does not affect N-cadherin gene expression. E- and N- cadherin undergo sequential cleavage, or ‘cadherin shedding’ at the plasma membrane (Supplementary Figure S5A). ADAM10 cleaves the ∼ 80 kDa ectodomain of N-cadherin resulting in its release into the extracellular medium. The C-terminal domain is subsequently cleaved into smaller fragments by the sequential action of presenilin-1 and caspase-3. If the reduction of full length N-cadherin was due to increased proteolysis at the plasma membrane, it would be expected that there would be a corresponding increase of C-terminal fragments of N-cadherin. However no such increase was observed in RCP-depleted cell lysates indicating that RCP is not involved in cadherin shedding at the plasma membrane (Supplementary Figure S5B). Also, treatment of siRNA transfected cells with a selective ADAM10 inhibitor, GI254023X, for 24 hours prior to lysis did not result in a recovery of full-length N-cadherin levels (Supplementary Figure S5C). Furthermore, RCP depletion did not consistently affect the overall level of the ADAM10 metalloproteinase, and actually resulted in a statistically significant decrease of presenilin-1 (Supplementary Figure S5D, S5E). These results demonstrate that the siRCP-induced reduction in N-cadherin levels is not caused by increased ‘cadherin shedding’ at the plasma membrane.

**Figure 5:**
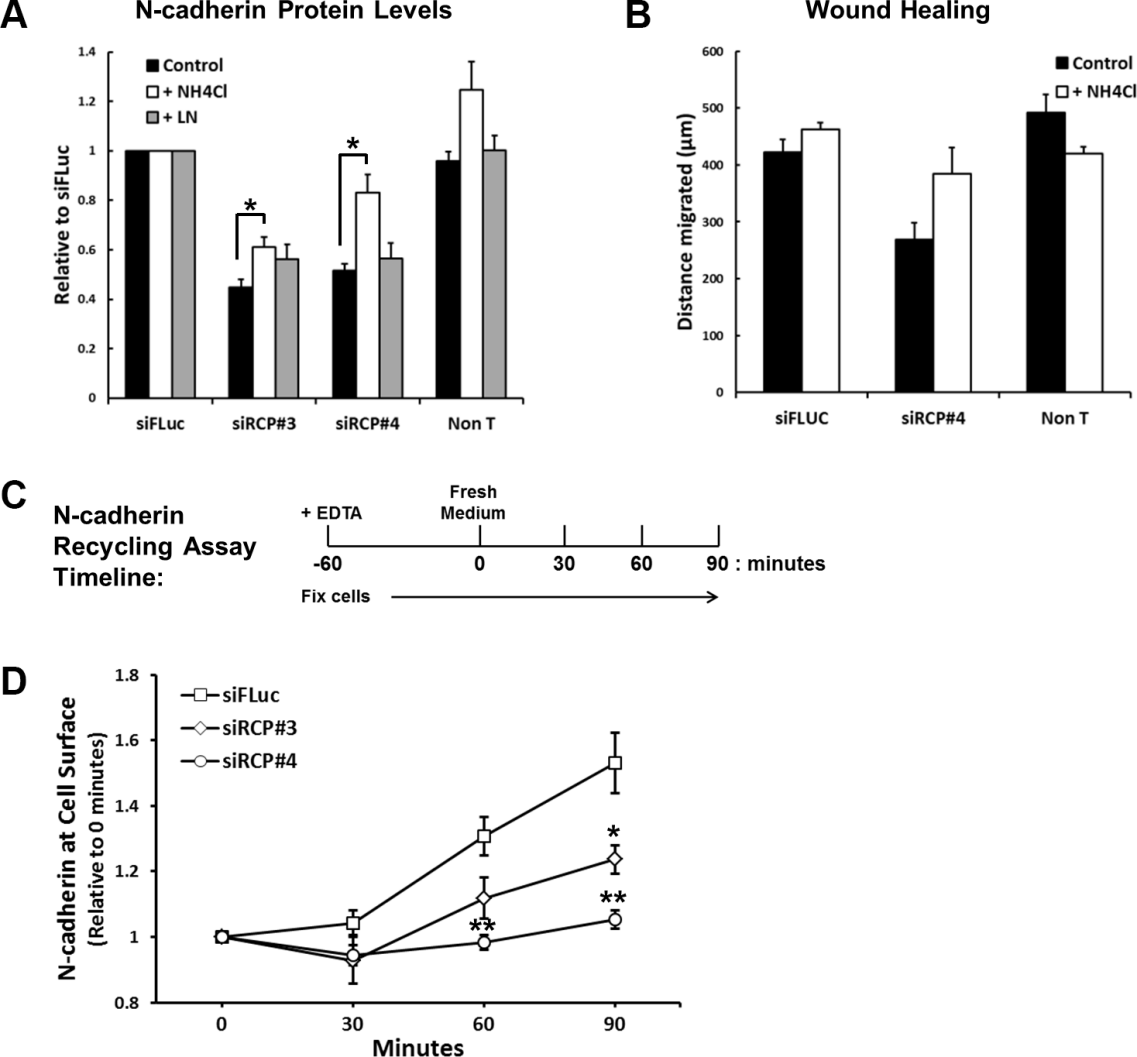
RCP regulates the recycling of endocytosed N-cadherin back to the plasma membrane (**A**) Histogram depicting the level of full-length N-cadherin protein in A549 cells transfected with the indicated siRNA duplexes for 72 hours and treated with vehicle alone, 20 mM NH_4_Cl, or 10 μM lactacystin for the final 24 hours (^*^p < 0.05; *n* = 3). (**B**) A549 cells were transfected with siFLuc or siRCP#4 for 72 hours. The monolayers were wounded and the fresh media was supplemented with or without 20 mM NH_4_Cl. The distance migrated is plotted in the histogram. (**C**) Timeline of the N-cadherin recycling assay (see Experimental Procedures). (**D**) N-cadherin recycling assay in cells transfected for 72 hours with the indicated siRNA duplexes (^*^p < 0.05, ^**^p < 0.01; *n* = 3).

To investigate whether the reduction of N-cadherin is due to increased lysosomal or proteasomal degradation, siRNA transfected cells were treated for 24 hours with NH_4_Cl, to inhibit lysosomal degradation, or lactacystin, a specific proteasomal inhibitor. Under these conditions a statistically significant recovery of N-cadherin levels was observed in the cells treated with NH_4_Cl (Figure 5A), indicating that RCP knockdown promotes the lysosomal degradation of N-cadherin. Lactacystin had no effect on N-cadherin (Figure 5A). NH_4_Cl treatment also led to a slight recovery in the migration of siRCP#4-transfected cells (Figure 5B).

These results suggest that RCP is involved in the recycling of endocytosed N-cadherin. In RCP-depleted cells the recycling of internalised N-cadherin to the cell surface is suppressed and is instead diverted to the degradative pathway where it is broken down in lysosomes. To determine if this is the case, we devised a quantitative N-cadherin recycling assay. In this assay, monolayers of siRNA transfected A549 cells are induced to internalise their adherens junction proteins by addition of the calcium chelator, EDTA, into the culture medium for 1 hour. The EDTA is washed out, fresh medium is added, and cell-cell junctions are allowed to reassemble for various times. At each time-point the cells are fixed and N-cadherin at the cell surface is detected and quantitated with an antibody that recognises its ectodomain (Figure 5C). Recycled N-cadherin is calculated by normalising cell surface N-cadherin at each time point to that at 0 hours (cells fixed immediately after EDTA washout). Even though there is a reduced amount of N-cadherin in RCP-depleted cells, there is still sufficient remaining at the cell surface to monitor its internalisation and recycling. We observed a significant inhibition of N-cadherin recycling in RCP-depleted cells in comparison to control cells (Figure 5D). Taken together, these data demonstrate that RCP functions to re-route endocytosed N-cadherin away from the degradative pathway and send it back to the plasma membrane.

**Figure 6:**
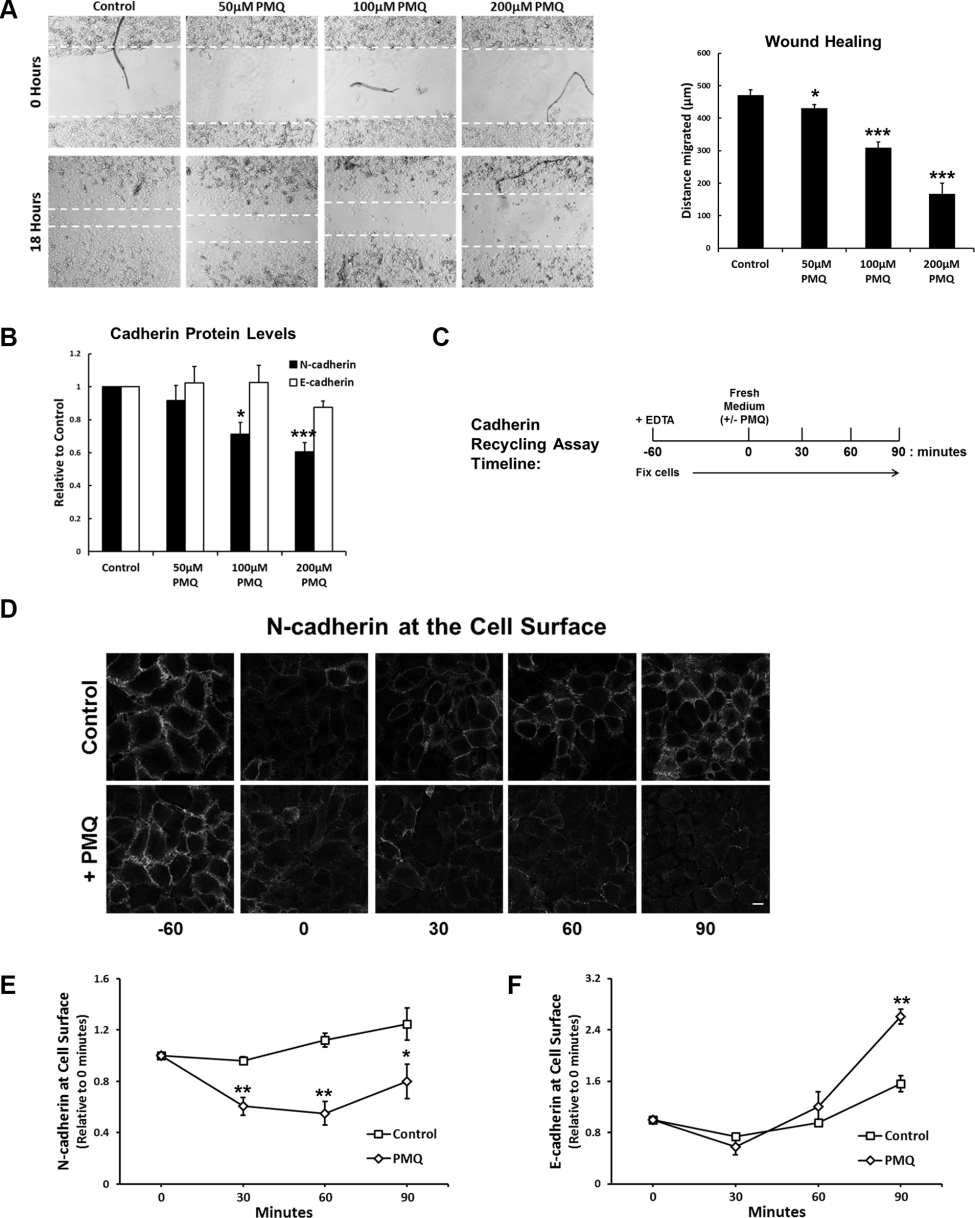
The endosomal recycling inhibitor primaquine mimics RCP knockdown (**A**) A549 cell monolayers were wounded and allowed to recover for 18 hours in media supplemented with the indicated concentrations of primaquine. The histogram depicts the distance migrated (^*^p < 0.05, ^***^p < 0.001; *n* = 4). (**B**) Protein levels of N-cadherin (black) and E-cadherin (white) in lysates from cells used in the wound healing assays above (^*^p < 0.05, ^***^p < 0.001; *n* = 4). (**C**) Timeline of the N-cadherin recycling assay used in C, D and E. (**D**) A549 cells treated as indicated in C, were fixed and N-cadherin at the cell surface was labelled with an antibody that recognised the N-cadherin ectodomain. (**E**) N-cadherin recycling assay in A549 cells allowed to recover in the presence or absence of 200 μM PMQ (^*^p < 0.05, ^**^p < 0.01; *n* = 4). (**F**) E-cadherin recycling assay in A549 cells allowed to recover in the presence or absence of 200 μM PMQ (^**^p < 0.01; *n* = 4).

To further investigate if N-cadherin undergoes endosomal recycling we made use of the endosomal recycling inhibitor, primaquine (PMQ). We found that PMQ inhibits wound healing in a dose-dependent manner (Figure 6A), and overnight treatment with PMQ leads to a reduction in N-cadherin protein levels without affecting E-cadherin levels (Figure 6B). In recycling assays, in which PMQ was added to the culture medium after the EDTA wash-out (Figure 6C), N-cadherin recycling was significantly inhibited (Figure 6D, 6E). Surprisingly, E-cadherin recycling was increased in the presence of PMQ (Figure 6F). These results demonstrate that endocytosed N-cadherin is returned to the plasma membrane along the endosomal recycling pathway.

**Figure 7:**
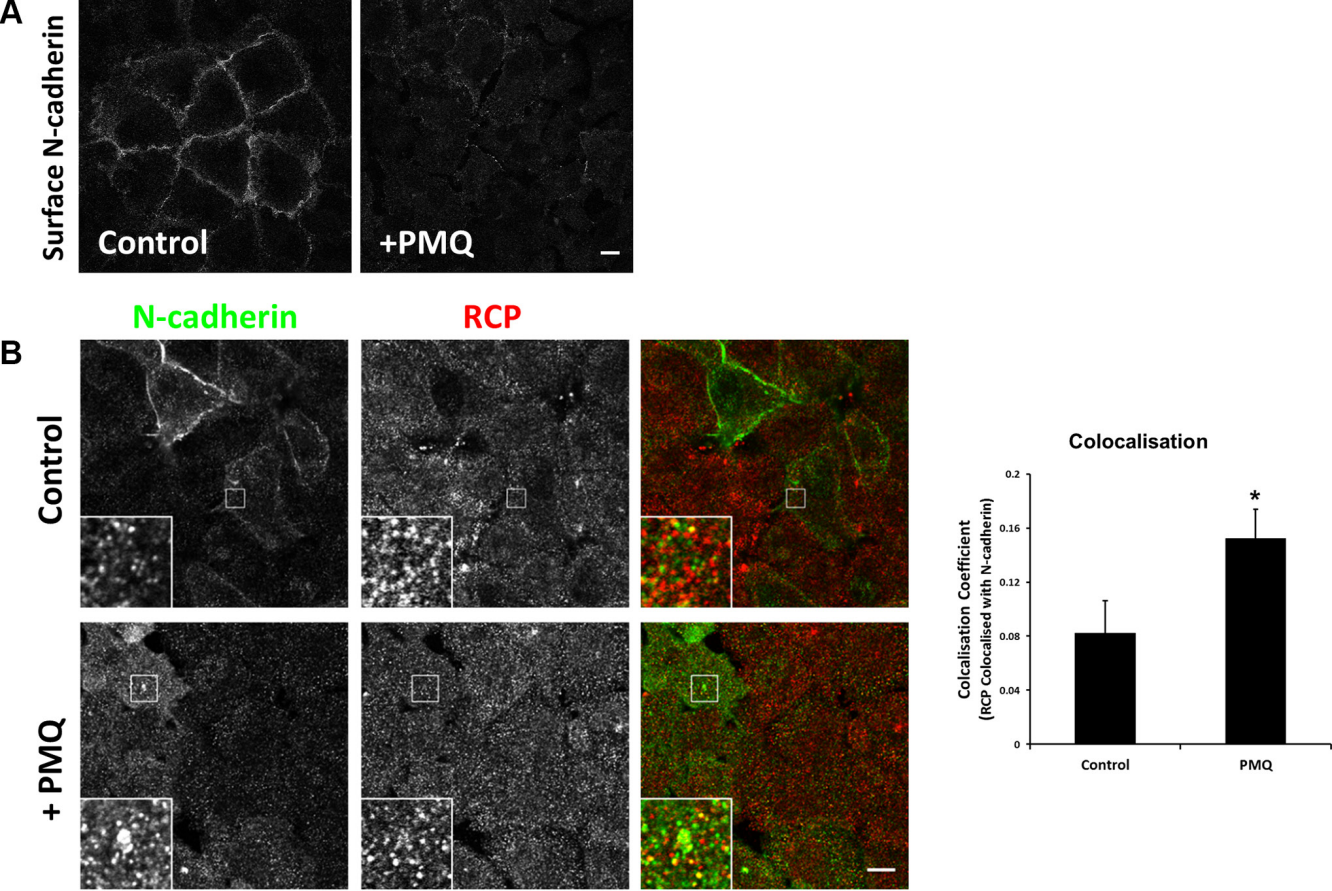
N-cadherin is continuously internalised and recycled in A549 cells (**A**) An A549 cell monolayer was treated +/− 300 μM PMQ for 1 hour at 37^°^C. The cells were fixed, quenched and N-cadherin at the plasma membrane was immunolabelled with an antibody that recognises its extracellular domain. Bar, 10 μm. (**B**) A549 cells, treated as above, were fixed, quenched and permeablised prior to co-staining with antibodies to N-cadherin (green) and RCP (red). Bar, 10 μm. The histogram depicts the degree of colocalisation between RCP and N-cadherin (^*^p < 0.05; *n* = 3).

Immunofluorescence labelling of non-permeablised A549 cells, pre-treated with 300 μM PMQ for 1 hour, with an antibody to the N-cadherin ectodomain revealed a significant reduction of cell surface N-cadherin in comparison to untreated cells (Figure 7A). This indicates that N-cadherin is continuously internalised and recycled, and PMQ traps the internalised N-cadherin inside the cell. PMQ treatment for 1 hour also resulted in a 1.8-fold increase in colocalisation between RCP and N-cadherin in intracellular vesicles (Figure 7B).

## DISCUSSION

### RCP plays a role in the migration of lung adenocarcinoma cells

The endosomal recycling pathway mediates the return of endocytosed proteins and lipids to the cell surface. It is one of the main cellular mechanisms for controlling the composition of the plasma membrane. It is emerging that aberrant expression of key regulators of endosomal recycling can lead to increased aggressiveness in a wide range of cancers, presumably due to downstream effects on cell signalling and the biased recycling of adhesion molecules, matrix metalloproteinases, and receptor tyrosine kinases [1, 2]. One such regulator is RCP, a membrane associated protein that binds to Rab GTPases of the endosomal recycling pathway (Rabs 4, 11, and 14) [10, 11, 31]. The RCP gene is amplified in a wide range of cancers [4, 15–18], and its overexpression has been linked to increased aggressiveness in breast cancer [5].

Furthermore, Caswell et al. have demonstrated that RCP drives the transport of α_5_β_1_ integrin and the EGF receptor to the leading edge of migrating ovarian cancer cells [6]. Here we demonstrate that RCP also plays a role in the motility and invasion (key features of metastatic tumour cells) of lung adenocarcinoma cells. RNAi-mediated downregulation of RCP led to an inhibition in the motility of these cells in cell migration assays. Conversely overexpression of wild-type RCP, but not an inactive mutant, increased cell motility. Cell migration is a critical process for tissue organisation during development and its dysregulation can lead to a number of diseases, including cancer.

### RCP regulates cadherin levels and influences epithelial-mesenchymal transition

In order to determine the molecular basis of the role of RCP in cell migration, we first examined if RCP knockdown affects the polarisation of migrating cells. The Golgi apparatus rapidly reorients itself forward of the nucleus and facing the leading edge in migrating cells [20]. We found that depletion of RCP disrupts this process, leading to a more random distribution of the Golgi in cells at the wound edge. Furthermore, the Golgi cisternae were much less compact in comparison to the control cells. Polarisation defects are usually associated with more random and less persistent cell migration, however analysis of cell trajectories in time-lapse movies of migrating cells revealed that RCP knockdown affected the velocity, but not the directionality of cells (Supplementary Figure S2C–S2E). This suggests that polarity defects cannot fully account for the observed inhibition in cell motility.

Given the accumulating evidence that Rab4, Rab11, and Rab14 are involved in E- and N- cadherin trafficking [13, 24, 25, 32, 33], we examined the effect of RCP knockdown on the localisation and expression of these cadherins. A reduction of N-cadherin protein levels was observed in cells depleted of RCP, by both immunofluorescence and Western blot. Surprisingly, this reduction in N-cadherin was accompanied by an increase in E-cadherin levels. In contrast, overexpression of wild-type RCP led to increased N-cadherin expression without affecting E-cadherin levels. E-cadherin and N-cadherin are important factors in the invasion and growth of epithelial cancer cells, with E-cadherin acting as a ‘brake’ on invasion while N-cadherin promotes invasion and metastasis [26, 27]. Indeed, N-cadherin is upregulated in the most invasive and de-differentiated breast and prostate cancers [34, 35]. An N-cadherin antagonist was able to suppress the enhanced migration of GFP-RCP_WT_ expressing cells. We hypothesise that the reduced levels of N-cadherin, and concomitant increase of E-cadherin, at the surface of RCP-depleted cells is a major contributor to their reduced motility.

The levels of E- and N- cadherin are normally tightly regulated and alterations in the balance of these two cadherins can lead to cells undergoing epithelial-mesenchymal transition (EMT). EMT is a process in which epithelial cells lose their characteristic polarity, disassemble cell-cell junctions and become more motile. This process is important for normal development, but also during tumour metastasis. One of the key features of EMT is cadherin switching in which cells shift from expressing E-cadherin to expressing N-cadherin. Cancer cells often recapitulate this switch, resulting in an aggressive tumour cell that can leave the primary tumour and metastasize [36]. The cadherin switch in A549 cells treated with the TGFβ1 ligand, to induce EMT, was dampened when RCP was knocked down. This data suggests that RCP can directly influence EMT. EMT is also associated with morphological changes, in which cells transform from a typical epithelial ‘cobblestone’ appearance to a more loosely connected elongated appearance with prominent actin stress fibres. Induction of RCP overexpression in the A549 stable cell lines was sufficient to trigger these cells to adopt a mesenchymal morphology. These results indicate that RCP overexpression can promote EMT, and that depletion of RCP results in cells assuming a more epithelial and less motile phenotype.

### RCP regulates N-cadherin endosomal recycling

Cadherin expression in epithelial tumours can be regulated at both transcriptional and post-transcriptional levels. RT-qPCR revealed that RCP knockdown did not consistently alter E- or N- cadherin mRNA levels, suggesting that RCP regulates cadherin expression at the post-transcriptional level. E- and N- cadherin undergo proteolytic processing at the plasma membrane by the sequential action of a number of proteases including ADAM10, presenilin-1, and caspase-3. The ADAM10 metalloproteinase cleaves the ectodomain of both E- and N- cadherin at the plasma membrane, releasing it into the extracellular environment in a process called “shedding”. The intracellular domains of the cadherins are further cleaved by presenilin-1 and caspase-3. Linford et al. have proposed that Rab14 plays a role in the transport of ADAM10 to the plasma membrane, thereby regulating the processing of N-cadherin [13]. However, our results indicate that RCP does not play a role in N-cadherin shedding as we do not observe an increase of N-cadherin C-terminal fragments in RCP-depleted cells, and an ADAM10 inhibitor does not restore the levels of full-length N-cadherin. Indeed, while we did observe that depletion of Rab14 inhibits cell motility (Supplementary Figure S6B, S6C); we could not observe an effect of Rab14 depletion on the level of N- or E- cadherin in A549 cell lysates (Supplementary Figure S6D–S6F).

**Figure 8:**
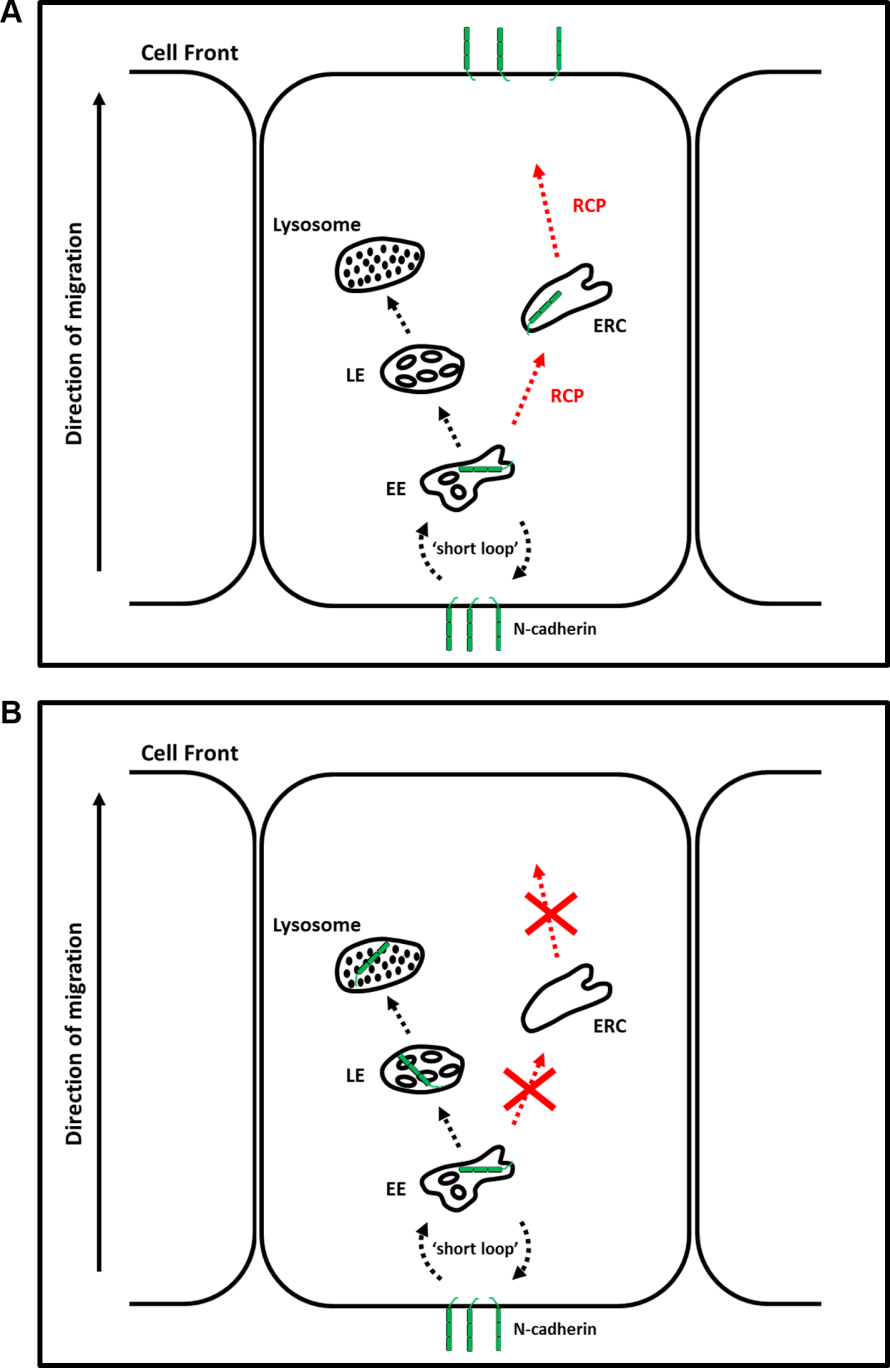
RCP regulates the endosomal recycling of N-cadherin (**A**) N-cadherin endocytosed from the cell rear undergoes RCP-dependent polarised recycling to the leading edge. (**B**) In the absence of RCP, N-cadherin is diverted to lysosomes where it is degraded. EE–Early Endosome; ERC–Endosomal Recycling Compartment; MVB–Multivesicular Body.

To investigate whether RCP-depletion led to increased lysosomal or proteasomal degradation of N-cadherin we treated siRNA transfected cells with ammonium chloride or lactacystin for the final 24 hours of the transfection. Ammonium chloride is a lysosomal inhibitor and lactacystin is a selective proteasome inhibitor. Lactacystin had no effect, but ammonium chloride treatment resulted in a 36–61% recovery of N-cadherin levels. This suggests that RCP-depletion leads to increased lysosomal degradation of N-cadherin and we propose a model in which RCP regulates the recycling of endocytosed N-cadherin to the plasma membrane (Figure 8A). In the absence of RCP, N-cadherin cannot be recycled and is diverted to the degradative pathway (Figure 8B).

The increase of E-cadherin protein levels in RCP-depleted cells may be due to a surplus of p120 catenin, since N-cadherin is downregulated in these cells. p120 catenin associates with both cadherins and binds to the juxtamembrane domain of E-cadherin masking a dileucine motif, thus preventing its endocytosis and leading to its stabilisation at the plasma membrane [37]. Depletion of RCP strongly inhibited the recycling of N-cadherin to the cell surface in a quantitative cadherin trafficking assay. N-cadherin has recently been found to undergo polarized recycling from the cell rear to the leading edge in migrating primary rat astrocytes [38]. The authors of this study demonstrate that N-cadherin undergoes clathrin-mediated endocytosis at the cell rear and is transported to the leading edge, in a process that takes 30–40 minutes. We found that the endosomal recycling inhibitor, primaquine, also significantly inhibited N-cadherin recycling in A549 cells. Furthermore, primaquine inhibited the migration of A549 cells and led to a reduction in N-cadherin, but not E-cadherin, protein levels. These results mimic the effects of RCP depletion and further confirm the importance of the endosomal recycling pathway in the transport of N-cadherin to the plasma membrane.

Interestingly, while this manuscript was under revision Boulay et al. reported that RCP plays a role in E-cadherin trafficking [39]. The authors reported that loss of RCP leads to the downregulation of E-cadherin and increased metastatic potential of ErbB2 positive breast tumours. This is the reverse of our observations in A549 lung adenocarcinoma cells, i.e. that loss of RCP leads to an increase of E-cadherin and a corresponding decrease in their metastatic potential. However, as the authors of this study point out, the ability of RCP to act as an oncogene or tumour suppressor is likely to be highly dependent on tumour type. Indeed, Rab25 which interacts directly with RCP has been found to act as an oncogene in ovarian cancer and certain breast cancers and as a tumour suppressor in colon and intestinal carcinomas [3].

The results presented here provide further evidence for the importance of the endosomal recycling pathway in epithelial cell carcinogenesis, by driving the transport of adhesion proteins and receptor tyrosine kinases to the leading edge of migrating tumour cells and helps to explain how aberrant expression of regulators of this pathway can lead to the increased aggressiveness of a wide range of cancers.

## MATERIALS AND METHODS

### Reagents and antibodies

General laboratory chemicals were from Sigma-Aldrich. Exherin (ADH-1) was from AdooQ Bioscience. The following antibodies were used; α-tubulin (mouse B-5-1-2; Sigma-Aldrich), GM130 (mouse clone 35; BD Biosciences), E-cadherin (mouse clone 36; BD Biosciences), E-cadherin (goat S-17; Santa Cruz), N-cadherin (mouse clone 32; BD Biosciences), N-cadherin (sheep #AF6426; R&D Systems), N-cadherin (rabbit #ab18203; Abcam), Rab14 (rabbit #R0656; Sigma) and RCP (chicken #GW21574A; Sigma). Secondary antibodies raised in donkey to chicken, goat, mouse, rabbit, and sheep conjugated to IRDye 700, IRDye 800CW, Alexa-488, and Cy3 were from Molecular Probes, Jackson ImmunoResearch, LI-COR, and Rockland. Texas Red-Phalloidin was from Molecular Probes.

### Cell culture and transfections

A549 cells were obtained from the ECACC and cultured in Dulbecco's modified Eagle's medium (DMEM) supplemented with 10% foetal bovine serum (Sigma), 100 U/ml penicillin/streptomycin, and 2 mM glutamine at 37^°^C in 5% CO_2_. For siRNA transfections Lipofectamine RNAiMax (Invitrogen) was used according to the manufacturer's instructions. RNAi duplexes were obtained from Sigma (siFLuc 5′- CUUACGCUGAGUACUUCGA −3′; siRCP#3 5′- CAAACAGAAGGAAACGAUA −3′; siRCP#4 5′- CCAUCAUCAGUGAGAACUU −3′; and siRCP#5 5′- CCUCGCUUCUGGAGUGUUA −3′). The stable cell lines were generated by transfecting A549 cells with pEGFP-C3, pEGFP-C3 RCP_WT_, and pEGFP-C3 RCP_I621E_ [31] using TurboFect (Thermo Fisher Scientific). Transfectants were selected with 800 μg/ml G418 for approximately two weeks and stable colonies were isolated using cloning rings. Fusion protein expression was induced by supplementing the culture medium with 5 mM sodium butyrate for 24 hours.

### Cell migration assays

For the wound healing assays, A549 cells in 24-well plates were scratched with a 200 μl pipette tip, washed twice with PBS and fresh culture medium was added. Brightfield images were recorded (0 hours) and the cells were returned to 37^°^C for 18 hours, after which another brightfield image was acquired. The distance that the wound front had migrated was calculated using ImageJ (NIH). The cells were subsequently lysed in 100 μl RIPA Buffer (10 mM Tris, pH 7.4; 1 mM EDTA; 5 μM EGTA; 1% TX-100; 0.1% sodium deoxycholate; 0.1% SDS; 140 mM NaCl) supplemented with protease inhibitor cocktail (Roche), in order to assess knockdown efficiency by Western blot.

For the live wound healing assays, A549 cells in 4-well plates were transfected for 72 hours with the indicated siRNA duplex. The monolayer was wounded with a 200 μl pipette tip, washed twice with PBS and fresh culture medium, supplemented with 25 mM Hepes pH7.4, was added. Plates were placed in an Olympus IX51 inverted microscope in a temperature controlled environmental chamber (Solent Scientific), set at 37^°^C. The plates were allowed to equilibrate for 30 minutes, following which brightfield time-lapse movies were recorded with a 10 × 0.3 NA air objective at 1 frame every 10 minutes for over 10 hours. Greater than 50 cells per condition were tracked using the Manual Tracking plugin of ImageJ and trajectories, velocities and directionalities were calculated using the Chemotaxis and Migration Tool Version 2 (Ibidi).

For the migration assay, knockdown cells or cells stably expressing the GFP-fusion proteins, were detached with trypsin, counted and 30,000 cells were seeded per well (in duplicate) in serum-free medium into the upper chamber of a CIM-16 Transwell plate (Acea Biosciences). Serum-containing medium was used as the chemoattractant in the lower chamber. The plate was placed in an xCelligence RTCA DP Analyzer located in a tissue culture incubator and Cell Index (CI) measurements were recorded every 30 minutes for 72 hours.

The invasion assay was performed exactly as above, with the exception that the A549 cells were seeded on top of a layer of Matrigel (BD Biosciences) in the upper chamber.

### Endothelial monolayer invasion assay

The intravasation assay was performed as described previously [40]. In brief, 2.5 × 10^4^ HUVEC cells, in 100 μl EGM-2 medium (Lonza), were seeded in each well of an E-16 plate (Acea Biosciences) that had been pre-coated with 0.1% gelatin. The plate was placed in the xCelligence RTCA DP Analyzer and the endothelial cells were allowed to form a monolayer. Once the impedance readings reached a plateau (∼ 18–21 hours), A549 cells were detached with 5 mM EDTA/PBS, washed twice with PBS, and 10,000 cells in 100 μl complete DMEM was added to each well. Impedance readings were recorded every 10 minutes for 7 hours.

### Immunofluorescence microscopy

Cells were seeded on 10 mm glass coverslips, fixed with 4% paraformaldehyde, quenched with 50 mM NH_4_Cl, and blocked/permeablised with 0.05% saponin/0.2% bovine serum albumin. Cells were incubated with the indicated primary antibodies in the blocking solution. The secondary antibodies used were Cy3-conjugated donkey anti-rabbit from Jackson ImmunoResearch Laboratories (West Grove, PA) and Alexa^488^–conjugated goat anti-chicken from Molecular Probes. The cells were washed extensively with phosphate-buffered saline (PBS) between antibody incubations, and the coverslips were mounted in Mowiol. To immunolabel cell surface cadherin the cells were blocked with 1% BSA/PBS and all antibody incubations were performed in 1% BSA/PBS.

Images were acquired on a Zeiss LSM510 confocal microscope (Carl Zeiss, Jena, Germany). Colocalisation coefficients were calculated using the colocalisation module of the Zeiss Zen 2009 software.

### Cadherin recycling assays

A549 cells were seeded in 96-well plates and allowed to form monolayers. The cells were induced to internalise their adherens junctions by incubating with 2.5 mM EDTA in 100 μl serum-free medium for 1 hour. The cells were then washed twice with PBS and 100 μl fresh complete medium was added and cell-cell junctions were allowed to re-form for 0, 30, 60, and 90 minutes at 37^°^C. The cells were fixed at each time-point by addition of 100 μl 8% paraformaldehyde, directly into the culture medium. After quenching with 50 mM NH_4_Cl, the cells were blocked with 1% BSA/PBS for 1 hour at room-temperature (RT). E- and N- cadherin at the cell surface was labelled with goat anti-E cadherin (Santa Cruz) and sheep anti-N cadherin (R&D Systems) antibodies in 1% BSA/PBS, respectively. The wells were extensively washed with PBS and incubated with donkey anti-goat or anti-sheep conjugated to IRDye 800 CW plus DRAQ5 (Abcam) in 1% BSA/PBS for 1 hour at RT. The cells were extensively washed with PBS and scanned on an Odyssey Infrared Imager (Licor). The cadherin signal in each well was normalised against the DRAQ5 far-red fluorescent DNA dye signal.

## SUPPLEMENTARY MATERIALS FIGURES


