# Supplementary material for: Rab coupling protein mediated endosomal recycling of N-cadherin influences cell motility
Source: Oncotarget. 2016 Jul 9;8(62):104717–32. doi: 10.18632/oncotarget.10513 (PMC5739595; doi:10.18632/oncotarget.10513)
Supplement: Supplementary file 1 [file oncotarget-08-104717-s001.pdf]

Rab coupling protein mediated endosomal recycling of N-cadherin influences cell motility

Supplementary Materials

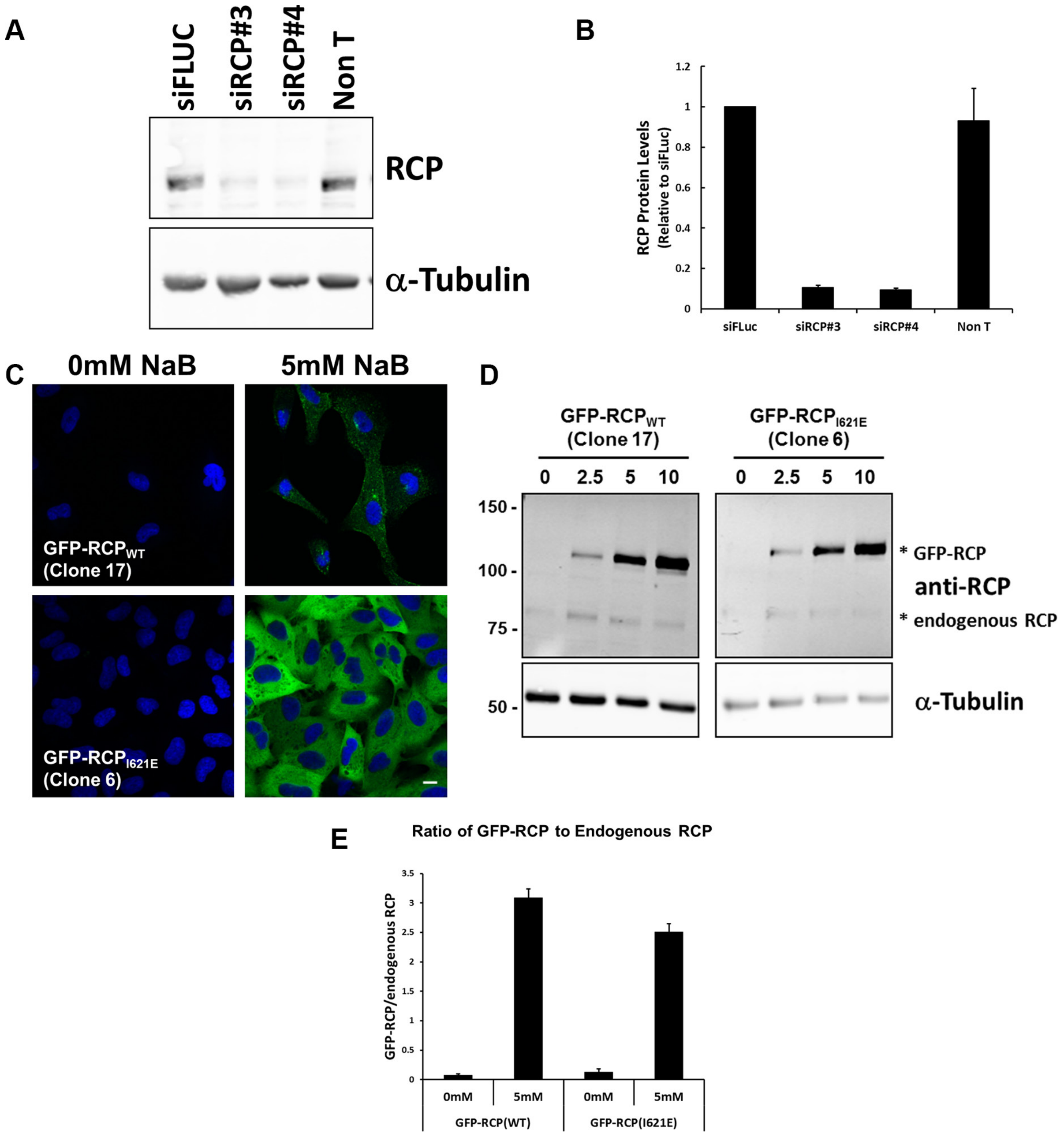

Supplementary Figure S1: Characterisation of RCP knockdowns and RCP stable cell lines. (**A**) Representative Western blot of RCP in lysates generated from cells transfected with the indicated siRNA duplexes for 72 hours, α-tubulin is used as a loading control. (**B**) Quantification of RCP protein levels in A549 cells transfected for 72 hours with the indicated siRNA duplexes (n = 6). (**C**) Fluorescence micrographs of the stable cell lines used in this study, treated +/– 5 mM sodium butyrate for 24 hours prior to fixation and labelling with DAPI (blue). (**D**) Western blots of lysates from the stable cell lines treated with the indicated concentrations of sodium butyrate for 24 hours and probed with anti-RCP. α- tubulin is used as a loading control. (**E**) Quantification of the ratio of GFP-RCP to endogenous RCP in the stable cell lines treated +/– 5 mM sodium butyrate. Values are mean +/– S.E.M. (n = 3).

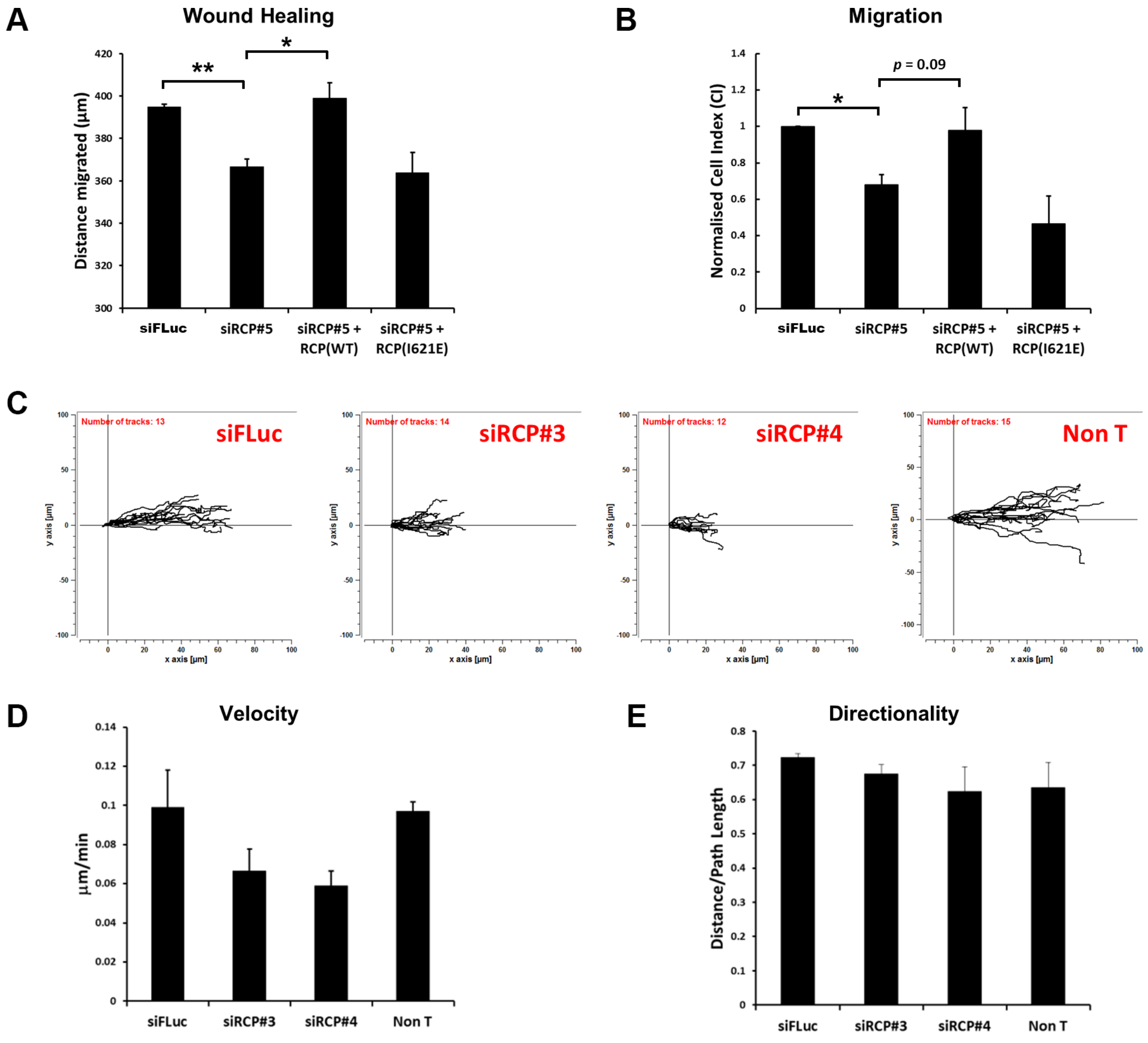

Supplementary Figure S2: Rescue of siRNA-mediated inhibition of cell motility. (**A**) The A549 RCP_{WT} and A549 RCP_{I621E} stable cell lines were transfected with siFLuc or siRCP#5 for 72 hours. The cell monolayers were wounded and brightfield images were recorded. The cells were returned to 37°C for 18 hours and imaged again. The distance migrated by the wound front is plotted in the bar graph. GFP-RCP_{WT} or GFP-RCP_{I621E} expression was induced by treatment with 5 mM sodium butyrate for the final 24 hours of the experiment. Error bars indicate the standard error of the means ($^{*}p < 0.05$, $^{**}p < 0.01$; $n = 3$). (**B**) The A549 RCP_{wt} and A549 RCP_{I621E} stable cell lines were transfected with siFLuc or siRCP#5 duplexes for 72 hours, detached and seeded, in duplicate, on CIM-16 Transwell plates and subjected to real-time migration assays (xCelligence). The histogram depicts the Normalised Cell Index (Cl) after 48 hours of migration. GFP-RCP_{WT} or GFP-RCP_{I621E} expression was induced by treatment with 5 mM sodium butyrate for 24 hours prior to seeding on the CIM-16 plate, and 5 mM sodium butyrate was maintained in the medium in the upper chamber of the plate for the duration of the migration assay. Error bars indicate the standard error of the means ($^{*}p < 0.05$; $n = 3$). (**C**) Representative trajectories of live A549 cells, transfected with the indicated siRNA duplexes, migrating into a wound for 10 hours (1 frame every 10 minutes). (**D**) Quantification of average velocity of the A549 cells extracted from > 50 track plots per condition, from 3 independent experiments. Values shown are mean ± SEM. (**E**) Quantification of directionality (distance travelled divided by path length) of the A549 cells extracted from > 50 track plots per condition, from 3 independent experiments. Values are mean ± SEM.

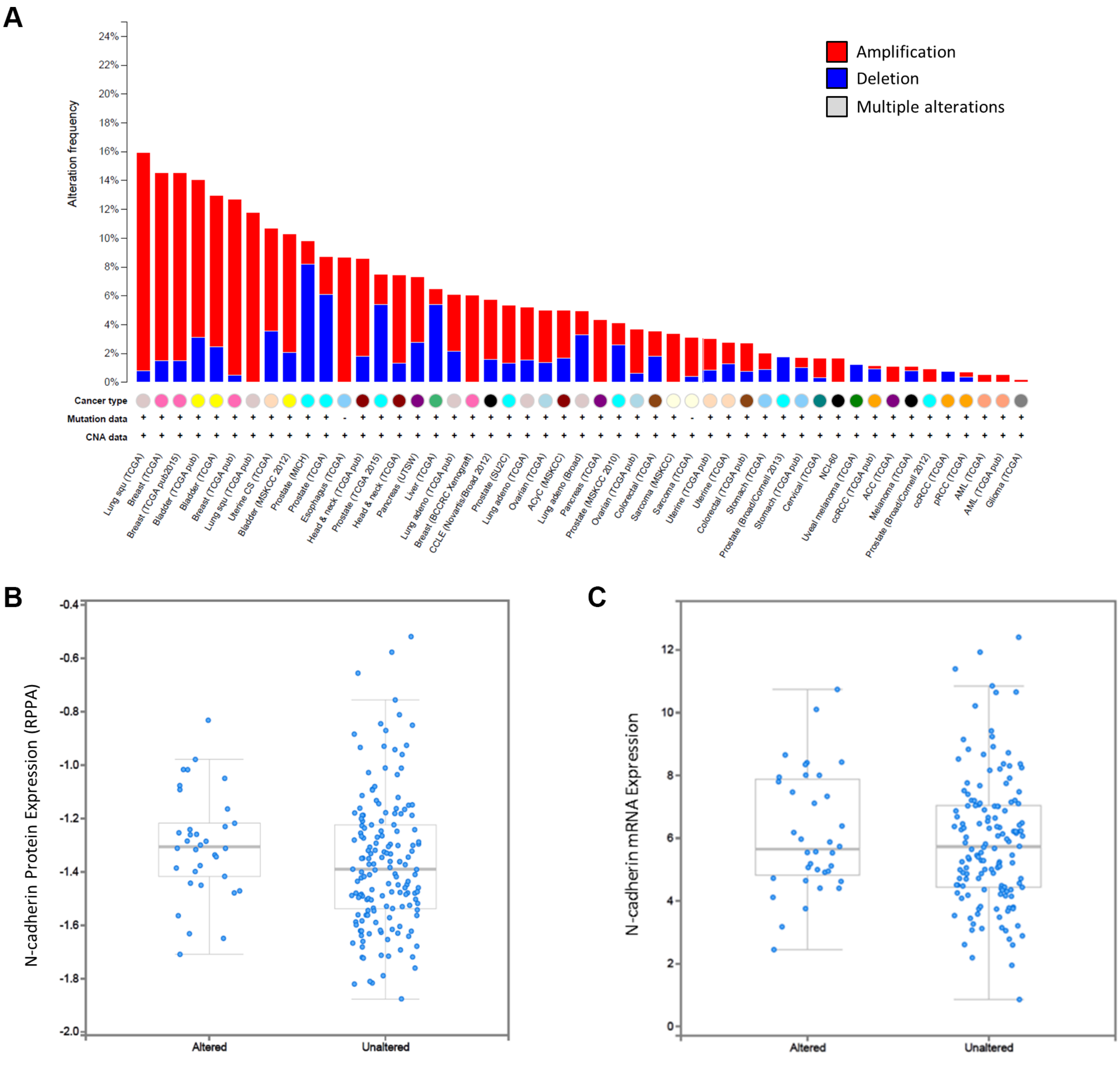

Supplementary Figure S3: The RCP gene is frequently altered in cancer. (**A**) Cross cancer histogram of the RCP gene in human cancer (cBioPortal). (**B**) N-cadherin protein expression in lung squamous cell tumours in which the RCP gene is altered or unaltered. (**C**) N-cadherin mRNA levels in lung squamous cell tumours in which the RCP gene is altered or unaltered.

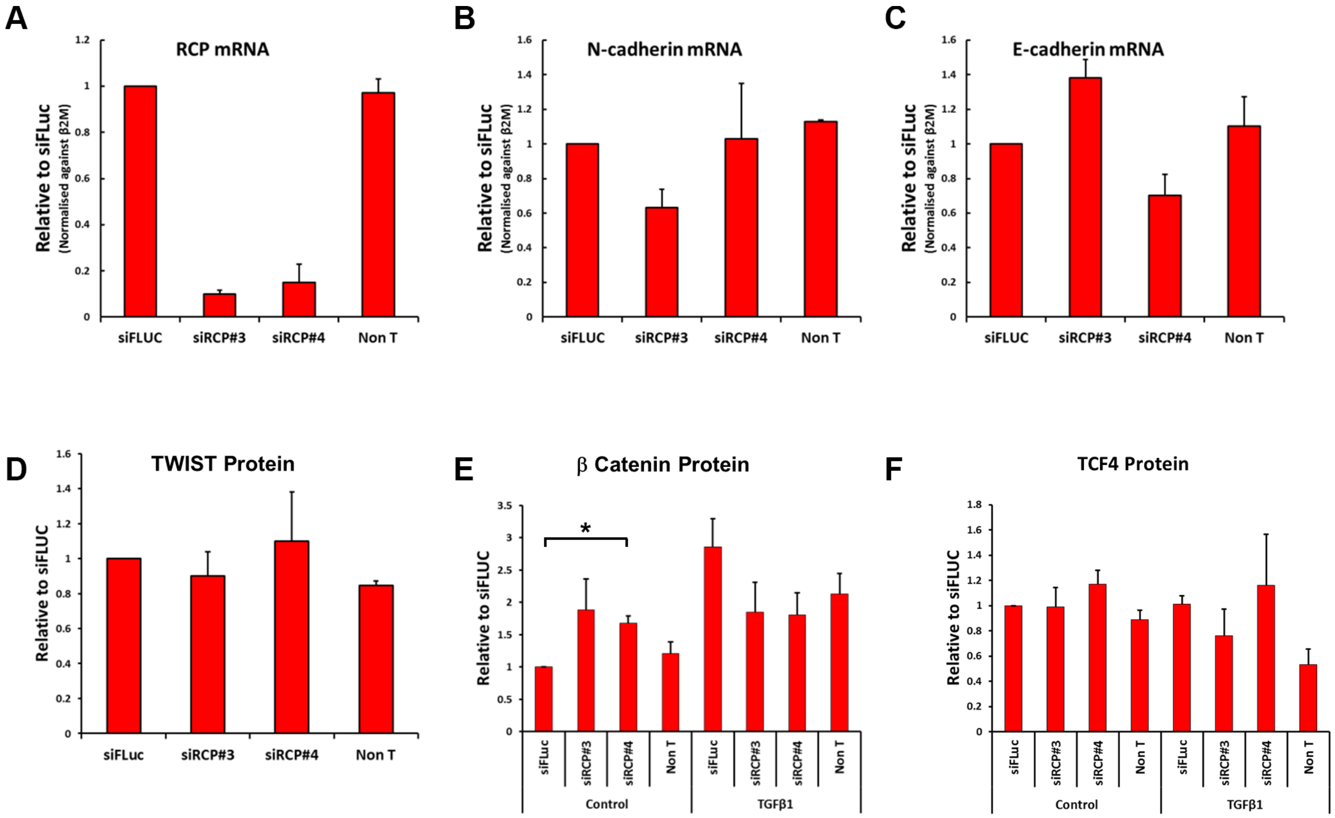

Supplementary Figure S4: Protein and mRNA levels in RCP knockdown cells. RCP (**A**) N-cadherin (**B**) and E-cadherin (**C**) mRNA levels (normalised to (β_2 microglobulin) in A549 cells treated with the indicated siRNA duplexes for 72 hours and assessed by RT-qPCR (n = 3). (**D**) Levels of TWIST protein in A549 cells transfected with the indicated siRNA duplexes for 72 hours. Values are mean ± S.E.M. (n = 3). Histogram indicating the levels of (β-catenin (**E**) and TCF4 (**F**) proteins in A549 cells transfected with the indicated siRNA duplexes for 24 hours and treated +/– 5 ng/ml TGFpi for a further 48 hours. Values are normalised to siFLUC Control ($^*p < 0.05$; n = 5).

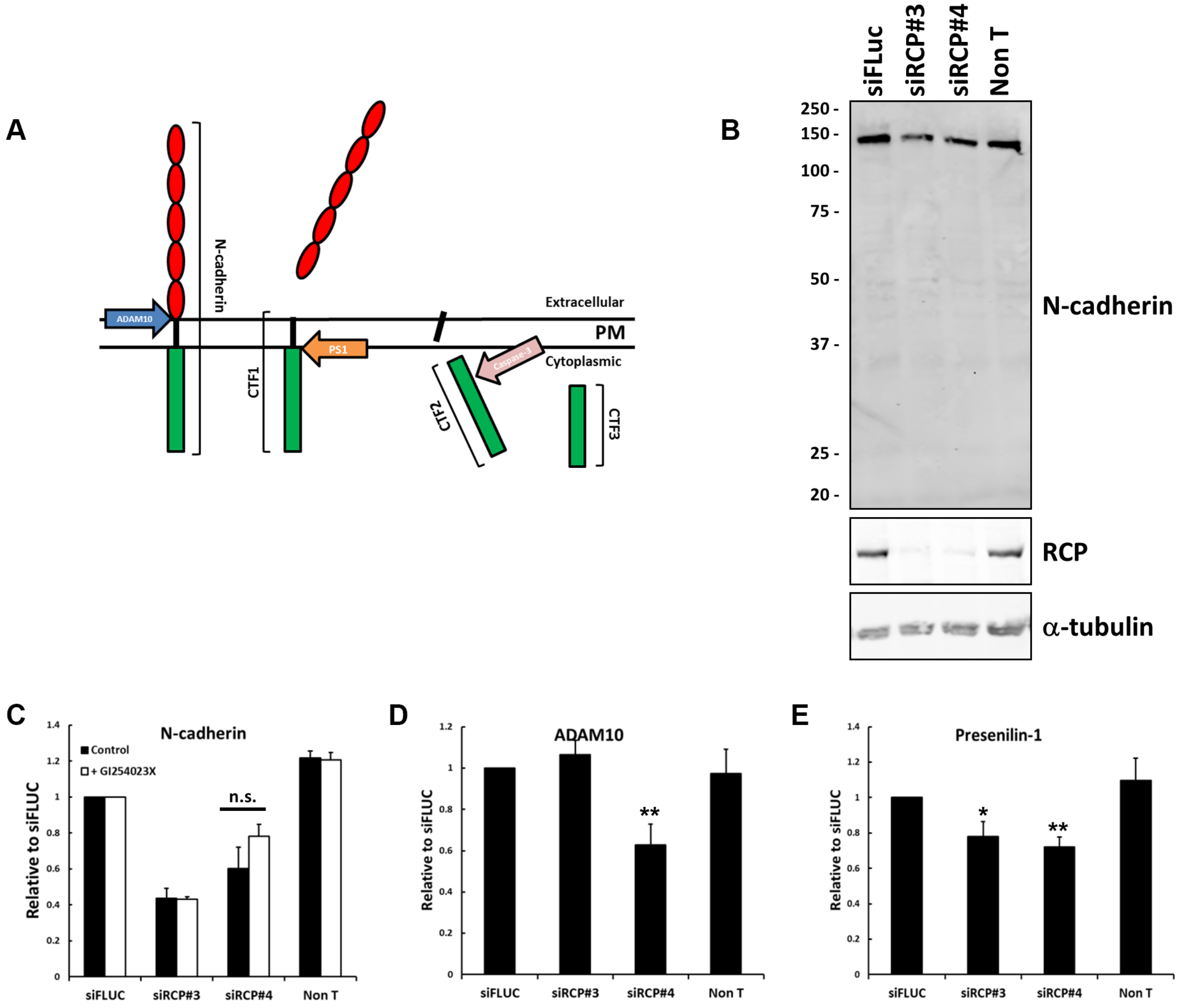

Supplementary Figure S5: RCP Knockdown Does Not Affect Processing of N-cadherin at the Cell Surface. (**A**) Schematic diagram depicting the proteolytic processing of N-cadherin that takes place at the plasma membrane. (**B**) Full length Western blot of lysates generated from cells transfected with the indicated siRNA duplexes for 72 hours and probed with an N-cadherin antibody that detects the C-terminus. (**C**) Quantification of N-cadherin protein levels in cells transfected with the indicated siRNA duplexes for 72 hours and treated with or without the ADAM10 inhibitor, GI254023X, for the final 24 hours (n = 3). Quantification of ADAM10 (**D**) and Presenilin-1(**E**) protein levels in A549 cells transfected with the indicated siRNA duplexes for 72 hours (n = 6).

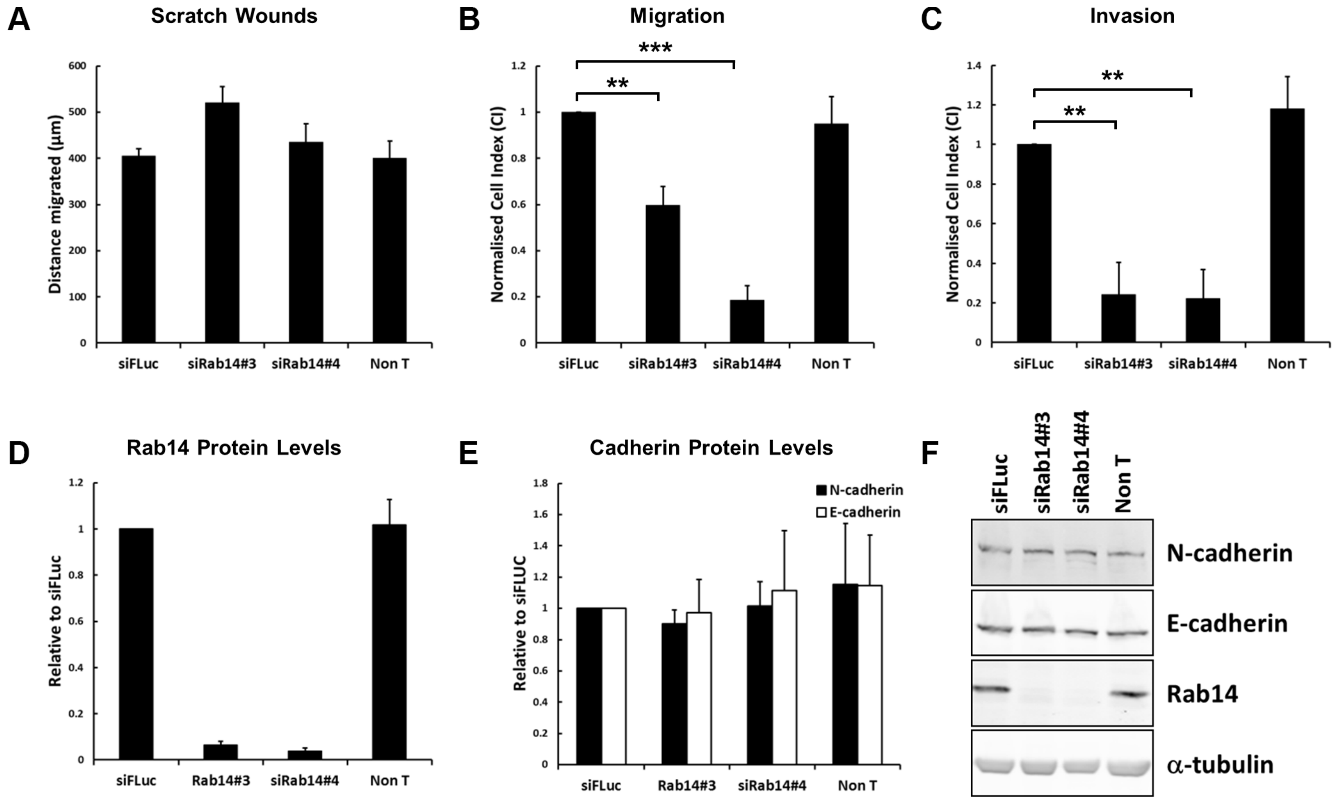

Supplementary Figure S6: Effect of Rabl4 knockdown on A549 cell motility and cadherin protein levels. (**A**) A549 cells in 24-well plates were transfected with a control siRNA or two siRNA duplexes targeting Rabl4. 72 hours post-transfection the cell monolayers were wounded and brightfield images recorded. The cells were returned to 37°C for 18 hours and imaged again. The distance migrated by the wound front is plotted in the bar graph. Error bars indicate the standard error of the means ($n = 3$). (**B**) A549 cells were transfected with the indicated siRNA duplexes for 72 hours, detached and seeded in duplicate on CIM-16 Transwell plates and subjected to real-time migration assays (xCelligence). The histogram depicts the Normalised Cell Index (Cl) after 24 hours of migration. Error bars indicate the standard error of the means ($^{**}p < 0.01$, $^{***}p < 0.001$; $n = 4$). (**C**) A549 cells transfected with the indicated siRNA duplexes for 72 hours, were detached and seeded, in duplicate, on CIM-16 Transwell plates that had been coated with Matrigel. The histogram depicts the Normalised Cell Index (Cl) after 48 hours of migration. Error bars indicate the standard error of the means ($^{**}p < 0.01$; $n = 3$). (**D**) Histogram represents the levels of Rabl4 protein in lysates from cells transfected with the indicated Rabl4 siRNA duplexes for 72 hours ($n = 3$). (**E**) Histogram represents the level of N-cadherin (black) and E-cadherin (white) protein in lysates from cells transfected for 72 hours with the indicated siRNA duplexes. Values are normalised to siFLuc ($n = 3$). (**F**) Representative Western blots of lysates from A549 cells transfected with the indicated siRNA duplexes for 72 hours, α-tubulin was used as a loading control.
